# Changing Beliefs in Managing Nausea Among Older Adults With Cancer From a Longitudinal Randomized Controlled Trial

**DOI:** 10.1093/geroni/igaf122.3200

**Published:** 2025-12-31

**Authors:** Kailei Yan, Arsham Alamian, Michael Owings, Victoria Loerzel

**Affiliations:** University of Central Florida, Orlando, Florida, United States; University of Miami, Coral Gables, Florida, United States; University of Central Florida, Orlando, Florida, United States; University of Central Florida, Orlando, Florida, United States

## Abstract

Chemotherapy-Induced Nausea and Vomiting (CINV) is one of the most feared side effects of chemotherapy, with more severe consequences in older adults, including dehydration, electrolyte imbalance [1], frailty [2], and poor adherence to treatments [3]. A perception gap exists regarding CINV management, with only 38% of patients fully complying with treatment, while clinicians underestimate CINV’s impact on patients’ daily lives [4]. Understanding beliefs in managing nausea is crucial to improving symptom management among older adults with cancer. This analysis examines the beliefs in managing nausea among older adults enrolled in a longitudinal, multicenter randomized controlled trial (RCT), evaluating Manage At Home (MAH), a novel serious game designed to help older adults self-manage CINV [5, 6]. The sample included in this analysis were 177 participants who completed baseline assessment, and 81 participants completed both baseline and the final assessment. Confirmatory factor analyses (CFA) were conducted to evaluate the construct validity of Symptom Representation Questionnaire (SRQ) [7], adapted to assess beliefs for both time 1 and final assessment. Results from CFA supported a four-factor structure—emotion, cure, time, and cause—at both baseline and final assessments, consistent with original instrument development [7]. However, Item 14 loaded onto multiple factors, suggesting potential ambiguity in wording. Linear mixed models indicated significant within-group improvements over time for all factors (p < .001), but no significant between-group differences over time, likely due to limited statistical power. MAH shows promise in improving beliefs related to self-management.

